# Immunophenotyping assessment in a COVID-19 cohort (IMPACC): A prospective longitudinal study

**DOI:** 10.1126/sciimmunol.abf3733

**Published:** 2021-08-10

**Authors:** IMPACC Manuscript Writing Team, on behalf of the IMPACC Network Steering Committee, Nadine Rouphael, Holden Maecker, Ruth R. Montgomery, Joann Diray-Arce, Steven H. Kleinstein, Matthew C. Altman, Steven E. Bosinger, Walter Eckalbar, Leying Guan, Catherine L. Hough, Florian Krammer, Charles Langelier, Ofer Levy, Kerry McEnaney, Bjoern Peters, Adeeb Rahman, Jayant V. Rajan, Steven Sigelman, Hanno Steen, Harm van Bakel, Alyssa Ward, Michael R. Wilson, Prescott Woodruff, Colin R. Zamecnik, Alison D. Augustine, Al Ozonoff, Elaine F. Reed, Patrice M. Becker, Nelson Agudelo Higuita, Matthew C. Altman, Mark A. Atkinson, Lindsey R. Baden, Patrice M. Becker, Christian Bime, Scott C. Brakenridge, Carolyn S. Calfee, Charles B. Cairns, David Corry, Mark M. Davis, Alison D. Augustine, Lauren I. R. Ehrlich, Elias K. Haddad, David J. Erle, Ana Fernandez-Sesma, David A. Hafler, Catherine L. Hough, Farrah Kheradmand, Steven H. Kleinstein, Monica Kraft, Ofer Levy, Grace A. McComsey, Esther Melamed, William Messer, Jordan Metcalf, Ruth R. Montgomery, Kari C. Nadeau, Al Ozonoff, Bjoern Peters, Bali Pulendran, Elaine F. Reed, Nadine Rouphael, Minnie Sarwal, Joanna Schaenman, Rafick Sekaly, Albert C. Shaw, Viviana Simon

**Affiliations:** ^1^Emory University, ^2^Stanford University, ^3^Yale School of Medicine, ^4^Boston Children’s Hospital-Harvard Medical School, ^5^Benaroya Research Institute, ^6^University of California-San Francisco School of Medicine, ^7^Yale School of Public Health,^8^Oregon and Health Sciences University, ^9^Icahn School of Medicine at Mount Sinai, ^10^Chan Zuckerberg Biohub, ^11^La Jolla Institute for Immunology, ^12^National Institutes of Allergy and Infectious Diseases/ National Institutes of Health, ^13^University of California- Los Angeles, ^14^Oklahoma University and Health Science Center, ^15^University of Florida, ^16^Brigham and Women’s Hospital, ^17^University of Arizona, ^18^Drexel University, ^19^Baylor College of Medicine, ^20^The University of Texas at Austin, ^21^Case Western Reserve University

## Abstract

The Immunophenotyping Assessment in a COVID-19 Cohort (IMPACC) is a prospective longitudinal study designed to enroll 1000 hospitalized patients with COVID-19 (NCT04378777). IMPACC collects detailed clinical, laboratory, and radiographic data along with longitudinal biologic sampling of blood and respiratory secretions for in-depth testing. Clinical and laboratory data are integrated to identify immunologic, virologic, proteomic, metabolomic, and genomic features of COVID-19–related susceptibility, severity, and disease progression. The goals of IMPACC are to better understand the contributions of pathogen dynamics and host immune responses to the severity and course of COVID-19 and to generate hypotheses for identification of biomarkers and effective therapeutics, including optimal timing of such interventions. In this report, we summarize the IMPACC study design and protocols including clinical criteria and recruitment, multisite standardized sample collection and processing, virologic and immunologic assays, harmonization of assay protocols, high-level analyses, and the data sharing plans.

## Teaser

IMPACC is a multicenter longitudinal study assessing clinical and immunologic manifestations of COVID-19 in hospitalized patients.

## INTRODUCTION

**Fig. 1.**
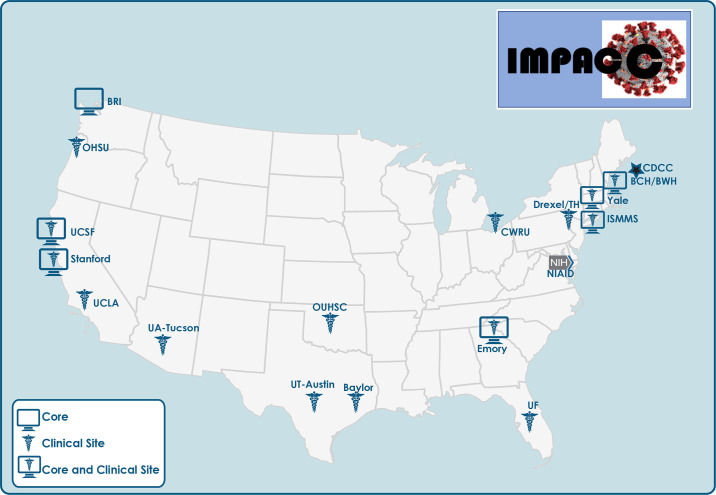
IMPACC sites and Core Labs. The 15 IMPACC clinical sites are located within 12 states across the United States. Core Labs are located at seven sites, six of which are co-located with clinical sites. Assays conducted by each Core Lab are indicated in Fig. 4.

The coronavirus disease 2019 (COVID-19) pandemic urgently demands comprehensive knowledge about the immunology, virology, and genetics of this disease caused by severe acute respiratory syndrome coronavirus 2 (SARS-CoV-2). Mobilization of the global scientific community has produced substantial translational findings with unprecedented speed but often from limited patient populations (*1*–*5*). The National Institute of Allergy and Infectious Diseases (NIAID), National Institutes of Health (NIH), launched a prospective longitudinal cohort study [Immunophenotyping Assessment in a COVID-19 Cohort (IMPACC)] in May 2020. IMPACC aims to enroll at least 1000 adults hospitalized for known or presumptive COVID-19 in about 20 hospitals associated with 15 U.S. biomedical research centers and collect clinical data and biological samples for up to 12 months after discharge. Harmonized clinical data are obtained and biologic samples are assayed at 11 centralized Core immunoassay laboratories (Fig. 1). The goal of the study is to better understand the contributions of the pathogen and host immune response in modulating the manifestations, severity, and course of COVID-19 and to identify potential biomarkers as well as inform therapeutic interventions. In this report, we summarize the overall study design, multicenter coordination and harmonization of clinical data and biologic sample collection and processing, protocols for virologic and immunologic core assays, and approaches for a high-level integrated analysis plan.

## STUDY DESIGN

### Study overview and rationale

IMPACC is an observational cohort study designed to survey clinical and immunologic manifestations of COVID-19 in hospitalized patients (NCT04378777), collaboratively developed by the NIAID and investigators from the Human Immunology Project Consortium (HIPC), Asthma and Allergic Diseases Cooperative Research Centers (AADCRC), and other NIAID-funded programs. The IMPACC network brings together expert clinicians, geneticists, and immunologists to assess the relationship between the clinical course and immune response to SARS-CoV-2 in racially, ethnically, and geographically diverse adult patient populations across the United States. IMPACC’s primary objectives are to (i) describe the relationship between specific immunologic assessments and severity of illness in hospitalized patients with COVID-19, controlling for time of illness onset, and concurrent participation in clinical trials or off-label use of investigational (or approved) therapeutic agents for COVID-19 and (ii) describe the relationship between burden of disease, assessed by duration of virus shedding in nasal secretions, and severity of illness in hospitalized patients with COVID-19. The study incorporates clinical data collection elements harmonized with the publicly available International Severe Acute Respiratory and Emerging Infection Consortium (ISARIC) case report forms (CRFs), as well as standardized biologic sample collection and processing protocols, to minimize confounding variables across sites. Local upper and lower airways and systemic immunologic parameters are surveyed via comprehensive, unbiased sample-sparing omics assessments (Supplementary Methods). An integrative data analysis plan is being developed to map immunologic endotypes (i.e., distinct functional subtypes of human immune responses) to clinical phenotypes in this patient cohort. In addition to focusing on unique features defining acute disease course, the cohort aims to follow participants for up to a year after hospital discharge to assess measures of both functional and immunologic recovery.

### Clinical study design

**Fig. 2.**
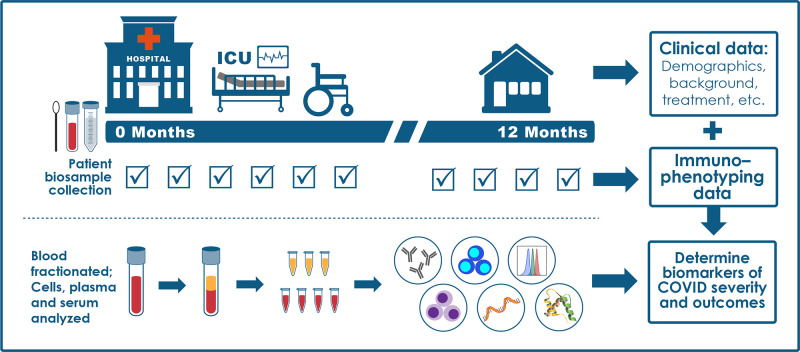
IMPACC study overview. This schematic represents the IMPACC study design in which clinical data, mid-turbinate nasal swabs, and blood samples are collected at each indicated visit during and after hospitalization. EAs are only collected from intubated patients.

The high-level study design is shown in Fig. 2. Participants are enrolled within 48 hours of hospital admission. Demographic information, COVID-19 symptoms and onset, and detailed medical history including comorbidities are collected from the medical record and/or patient interviews for all participants at baseline. Only cases with confirmed positive SARS-CoV-2 polymerase chain reaction (PCR) are followed longitudinally. Participants undergo extensive serial assessments to capture clinical data as shown in Box 1 (including clinical laboratory values, radiographic findings, medication use, oxygen and ventilatory support requirements, and complications) and biologic samples [blood, mid-turbinate nasal swabs, and, for intubated patients, endotracheal aspirates (EAs)]. Clinical data and samples are collected at enrollment and at days 4, 7, 14, 21, and 28 while participants are hospitalized. If a participant requires an escalation to intensive care unit (ICU)–level care or is discharged and readmitted to the hospital >48 hours after discharge, additional samples are collected within 24 and 96 hours of care escalation or readmission. Key clinical outcome data collected during hospitalization include mortality, level of care (floor, ICU), respiratory support requirements, extrapulmonary organ dysfunction, length of stay, and patient status based on an ordinal scale (*6*). If a participant is discharged from the hospital before day 14 or 28, attempts are made to collect additional data and samples at days 14 and 28 on an outpatient basis. Convalescent questionnaires and biologic samples are collected at 3-month intervals up to 12 months after hospital discharge.

For outpatient visits, both symptoms and functional recovery are surveyed using patient-reported outcome measures collected at the study site, by telephone, or via electronic data capture using an app or web portal. Specific patient-reported outcome measures to be assessed at outpatient follow-up include presence of COVID-19 symptoms, health-related quality of life using the EQ-5D-5L (*7*), and several PROMIS (*8*) surveys to capture physical, emotional, cognitive, psychosocial, and respiratory functional status. Efforts are made across sites to approach as many as possible hospitalized patients with known or confirmed COVID-19. Study information is provided to non–English-speaking patients in their native language.

NIAID staff conferred with the Department of Health and Human Services Office for Human Research Protections (OHRP) regarding potential applicability of the public health surveillance exception [45CFR46.102(l)(2)] to the IMPACC study protocol. OHRP concurred that the study satisfied criteria for the public health surveillance exception, and the IMPACC study team sent the study protocol and participant information sheet for review and assessment to institutional review boards (IRBs) at participating institutions. Concurrent enrollment in other IRB-approved observational or interventional protocols is allowed at the discretion of the investigators and local review boards. Most participating sites elected to conduct the study under the public health surveillance exclusion; several sites with previous IRB-approved biobanking protocols elected to integrate and conduct IMPACC under those protocols.

## MATERIALS AND METHODS

### Sample collection and processing

**Fig. 3.**
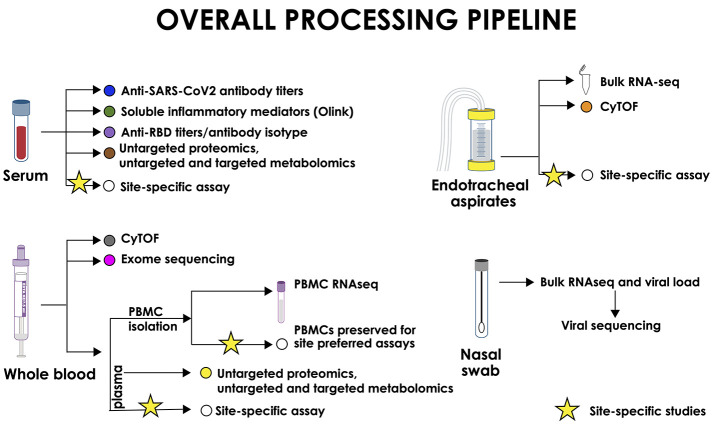
Sample processing pipeline and Core Lab assays. Nasal swabs are used to measure viral titers, for viral genome sequencing and metagenomics, and for bulk nasal transcriptomics. Serum samples are used to measure inflammatory markers (Olink), anti-SARS and human CoV antibodies, and for untargeted proteomics and untargeted/targeted metabolomics. Whole blood is used for genome-wide association study (GWAS), WES, and CyTOF. Plasma is used for untargeted proteomics and untargeted/targeted metabolomics. PBMCs are used for bulk transcriptomics analysis. EAs are processed for bulk transcriptomics and CyTOF analyses.

Sample collection was designed to meet minimal risk guidelines for blood collection for hospitalized adults, and sample-sparing assays are used when feasible. Blood samples (10 ml per time point) and nasal swabs (mid-turbinate) are collected at each specified time point, and blood is processed within 6 hours of collection according to the IMPACC standardized operating procedure. Whole blood and peripheral blood mononuclear cells (PBMCs) are collected to identify distinct immune cell populations and quantify changes in cell populations, gene expression, and activation markers [e.g., cytometry by time-of-flight (CyTOF) and bulk RNA transcriptomics] over the course of COVID-19 and convalescence. DNA is collected from whole blood at a single time point for genetic analyses (e.g., whole-exome sequencing). Serum is used to characterize SARS-CoV-2–specific antibodies, including virus neutralization, both serum and plasma are used for proteomics and metabolomics, and plasma is used to measure soluble inflammatory mediators (e.g., cytokines and chemokines) using oligonucleotide-linked antibody detection (Olink). RNA from the nasal swab is used to assess SARS-CoV-2 viral load and genomic sequence and to evaluate changes in immune-related upper airway epithelial gene expression (i.e., bulk transcriptomics). In addition, EAs are collected from intubated patients and processed within 2 hours of collection according to the IMPACC standardized operating procedures. EA cells are assessed by CyTOF and bulk transcriptomics to identify and quantify changes in gene expression and activation state of distinct immune cell populations in the lower respiratory tract. Processed samples are barcoded and centrally tracked on a laboratory information management system (LDMS; Frontier Science). All supplies necessary for sample collection and sample processing are centrally procured and supplied to the participating sites. Sample collection, processing, and storage procedures (Fig. 3) are standardized across sites, and samples are transported to centralized core laboratories (Core Labs) in batches for testing and analysis. The complete sample processing manual of procedures is included as Supplementary Methods.

Box 1. Demographic, clinical, laboratory, and radiographic assessments.

**Table:** 

1. Demographics 2. Targeted medical history 3. Outpatient and inpatient medications (including experimental medications, vasopressors, and neuromuscular blockade agents) 4. COVID-19 symptoms, symptom onset, and exposure history 5. Date of admission to hospital, date of admission or transfer to ICU (if applicable), and date of discharge 6. Targeted physical findings 7. Vital signs (temperature, heart and respiration rates, oxygen saturation) 8. Chest imaging findings 9. Laboratory findings a. CBC with differential b. Metabolic panel (to include serum creatinine, total bilirubin, liver function tests, and electrolytes) c. SpO_2_, arterial blood gas data d. PT/INR, D-dimer e. Ferritin, procalcitonin, LDH, CRP, cytokine panel f. Troponin, cardiac enzymes 10. Requirement for respiratory support a. New supplemental oxygen requirement (FIO_2_ and mode of delivery) b. Requirement for mechanical ventilation (include mode and settings, Pplat if available) c. Requirement for ECMO d. Use of prone positioning, inhaled nitric oxide 11. Requirement for new renal replacement therapy 12. Glasgow Coma Scale (GCS) 13. AVPU Scale (alert, verbal, pain, unresponsive) 14. Sequential Organ Failure Assessment Score (SOFA)

**Fig. 4.**
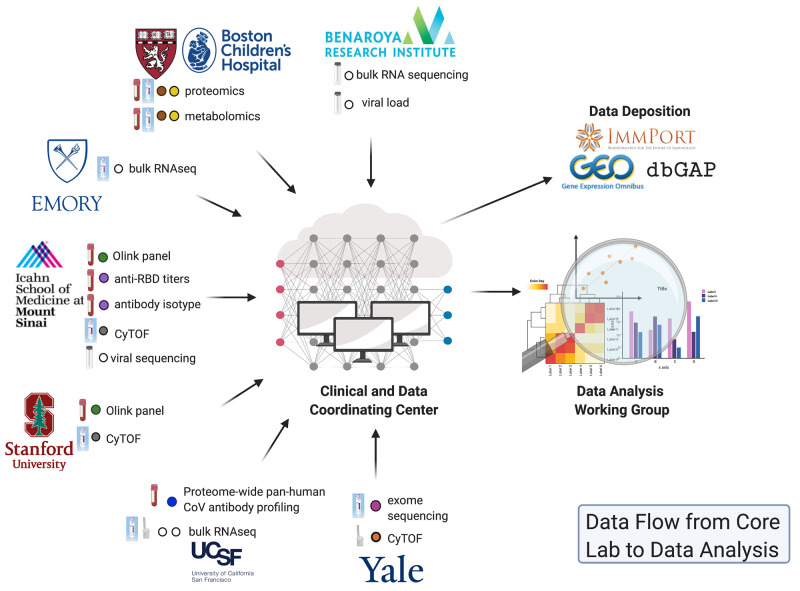
Data flow from Core Lab to data analysis. IMPACC Core Labs conduct the designated assays, QC, and preliminary data analysis before sending the validated datasets to the IMPACC CDCC for QA. After QC/QA, these data are provided to the DAWG for independent and integrated analyses to identify correlates of clinical outcomes, COVID-19 disease severity and progression, and multiomic signatures.

### Core Labs and technologies

IMPACC is using a systems immunology approach for simultaneous immune profiling on the same patient sample, using a wide range of assays, during COVID-19 disease and resolution. Profiling assays, which are sample sparing and provide a comprehensive, unbiased assessment of immunologic changes in the airways and circulation during disease progression and resolution, were chosen (Fig. 3). Core immune profiling laboratories have been established for in-depth sample analysis using optimized assays. The Core Labs work in close collaboration with the IMPACC clinical sites and their respective sample processing laboratories to ensure uniformity in sample collection and processing using the standardized manual of procedures. The shared methods and reagents promote high-quality assessment across all sample types. Each assay is performed at a single expert Core Lab or two harmonized Core Labs using rigorous standardized procedures, validated instruments and reagents, relevant controls, sample and batch randomization, assay timetables, and data sharing. The Core Lab assays have been chosen to provide comprehensive immune assessment while minimizing the amount of sample needed per assay.

#### Virus detection by real-time PCR assay

Viral shedding is increased and prolonged in severe compared with mild COVID-19 (*8*), but the precise relationship between viral kinetics and key clinical outcomes is not fully understood (*9*). Relating longitudinal kinetics of viral shedding to both host immune responses and clinical outcomes will provide important insights into mechanisms of severe disease. For patient comfort and consistency of collection, mid-turbinate swabs (rather than nasopharyngeal) are collected for serial viral quantification. Swabs are collected and placed in 1 ml of Zymo-DNA/RNA shield reagent (Zymo Research) and shipped to the Core Lab at Benaroya Research Institute (BRI). RNA is extracted using the Quick DNA-RNA MagBead Kit (Zymo Research) following the manufacturer’s instructions. Reverse transcription PCR (RT-PCR) for SARS-CoV-2 is performed on RNA extracts using the SARS-CoV-2 (2019-nCoV) CDC qPCR Probe Assay with target genes 2019-nCoV_N1, 2019-nCoV_N2, and control human RNase P (Integrated DNA Technologies) (*10*, *11*). Viral levels will be modeled longitudinally in relation to the kinetics of host immune responses and clinical outcomes.

#### Viral sequencing

During the global spread of SARS-CoV-2, several dominant lineages have emerged that are characterized by distinct mutation patterns and are tracked in related nomenclatures by Global Initiative on Sharing All Influenza Data (GISAID) (*12*) and others (*13*). Although most genetic changes in SARS-CoV-2 are expected to be neutral, selected mutations alter viral properties such as the ability to infect cells or evade host antibody responses (*14*–*16*). Treatment with antiviral drugs can also create selective pressure for the emergence of variants that escape drug effects. The availability of complete viral genomes for all participants will allow an assessment of host responses and outcomes in the context of specific SARS-CoV-2 strains and mutations. SARS-CoV-2 viral load will be assayed directly from mid-turbinate swab samples at each time point, and viral sequencing will be performed for the first nasal swab sample per participant with sufficient quantity of virus, at the Icahn School of Medicine at Mount Sinai (ISMMS; New York, NY). The team will use a combination of tiling primer designs to specifically amplify the SARS-CoV-2 genome and generate paired-end 2 × 150–nucleotide sequencing data on the Illumina platform to an average depth of >1000-fold. If needed, supplemental data may be generated on other platforms such as Ion Torrent to maximize the ability to obtain complete genomes for patients with low viral loads. The availability of deep sequencing data from each genome will also facilitate analyses of intrahost variants.

#### Serology

Antibody responses to SARS-CoV-2 are thought to be beneficial because they can neutralize viral entry and clear infected cells through effector functions. Antibodies may also protect from reinfection, although this hypothesis is still under investigation. However, the timing and magnitude of the antibody response have been linked to disease severity (*9*) and certain types of antibody responses may potentially be harmful, as has been hypothesized with regard to disease-enhancing antibodies in the context of dengue infection (*17*).

The IMPACC Serology Core Lab at ISMMS is quantifying the antibody response, including different isotypes, to the Spike protein of SARS-CoV-2 and against the receptor binding domain (RBD), which is the part of the Spike protein that interacts with angiotensin-converting enzyme 2 (ACE2)-immunosorbent assays (ELISAs) (*18*, *19*). ELISA titers will be reported as endpoint titers. Anti-spike and, especially, anti-RBD antibodies have been linked to virus neutralization. To assess the functional specificity of the antibody response, neutralization assays with authentic SARS-CoV-2 are being conducted (*20*). The readout for this assay is the 50% inhibitory dilution (ID_50_), which is calculated on the basis of virus neutralization compared with a negative control.

In addition to protein-based ELISAs, the IMPACC Serology Core Lab at UCSF is measuring serum antibody responses with a programmable phage display library [i.e., VirScan (*21*)] containing 38–amino acid overlapping peptides tiling across the SARS-CoV-2 and other human coronavirus (HuCoV) proteomes, including SARS-CoV-1 (NC_004718), beta coronavirus England 1 (NC_038294), HuCoV 229E (NC_002645), HuCoV HKU1 (NC_006577), HuCoV NL63 (NC_005831), HuCoV OC43 (NC_006213), Infectious Bronchitis virus (NC_001451), and Middle East respiratory syndrome–related coronavirus (MERS CoV) (NC_019843) (*22*). The VirScan assay measures the specificity of antibodies induced by SARS-CoV-2 infection, as well as HuCoV antibodies present early in the disease course likely as a result of past infections with other HuCoVs. These data may help determine whether the presence of preexisting HuCoV antibodies affects COVID-19 disease severity and whether specific aspects of the adaptive immune response to SARS-CoV-2 infection correlate with disease outcome. The oligonucleotides are cloned into T7 bacteriophage so that the viral peptides are displayed on the phage surface. The phage display library is then incubated with patient sera, and immunoglobulins are immunoprecipitated by magnetic protein A and G beads along with antibody-bound phage, which are sequenced to generate viral peptide counts whose fold enrichment is calculated relative to bead-only negative controls and prepandemic healthy control sera.

#### Serum cytokines

Selected proinflammatory cytokines in serum or plasma correlate with or even predict disease severity in COVID-19 (*1*, *23*, *24*). To comprehensively measure serum cytokines in a high-throughput manner for the IMPACC study, the Olink Inflammation Panel is used at the co-Core Labs at ISMMS and Stanford University. The Olink multiplex immunoassay offers the advantage of being a more comprehensive predictor of biological mechanisms at play than single cytokines (*1*). This method uses proximity extension assay (PEA) technology, whereby two antibodies for each target protein are conjugated to complementary oligonucleotides; if the correct antibody pair binds to the same target molecule, there is annealing of the oligos, and a PCR template can be created by extension and then dissociation of the extended product. Quantitative PCR is then carried out on the Fluidigm Biomark microfluidic platform. This platform allows for rapid setup of 96 samples × 96 reactions, with a minimal sample requirement, nominally 1 μl per sample. The inflammation panel consists of 92 analytes including pro- and anti-inflammatory cytokines, chemokines, and related molecules (https://olink.com/products/inflammation).

#### Proteomics and metabolomics

The blood serves a major role in modulating and distributing the immune responses throughout the entire body. Thus, determining how soluble immunomodulatory molecules are affected by SARS-CoV-2 infection (*25*) and recovery from COVID-19 is essential for a comprehensive understanding of immunophenotypes. To this end, selective quantitative maps of the plasma proteome and metabolome are being acquired by the Proteomics/Metabolomics Core (PMC) at Boston Children’s Hospital. To support the overarching goal of generating well-founded hypotheses to inform future research, unbiased liquid chromatography–mass spectrometry (LC-MS) methods are being used for proteomics and metabolomics. Our integrated sample-sparing proteomic and metabolomic workflow has been successfully used in populations with limited blood volumes (*26*).

The plasma proteomics analysis will take a two-pronged approach (*27*). First, the plasma proteome will be quantitatively mapped without any depletion in light of the important immunomodulatory roles of a sizable fraction of the standard depletion targets such as immunoglobulins and complement pathway components (*28*, *29*). Mapping the COVID-19–associated changes in abundance for these proteins is important for the comprehensiveness of the immunophenotyping efforts of IMPACC. This analysis will be performed in a high-throughput fashion using a triple quadrupole mass spectrometer operated in multiple reaction mode. We target 300 of the immunologically most relevant plasma proteins using a fast and highly sensitive state-of-the-art triple quadrupole mass spectrometer (LCMS-8060, Shimadzu). Then, the most abundant plasma samples will be depleted using biochemical methods that can be conducted in a high-throughput and cost-efficient manner on thousands of samples (*30*, *31*). The resulting depleted plasma samples are processed for analysis by LC-MS in discovery mode using a high-throughput sample delivery and high-performance liquid chromatography (HPLC) system (Evosep One) front-end and a Bruker ion mobility/quadrupole/TOF mass spectrometer (timsTOF Pro) back-end to ensure robustness.

The plasma metabolomics for the IMPACC study also follows a two-pronged approach. First, discovery metabolomics will be conducted in collaboration with Metabolon using reversed-phase LC-MS/MS in positive ion mode, reversed-phase LC-MS/MS in negative ion mode, and hydrophilic interaction liquid chromatography (HILIC) LC-MS/MS in negative ion mode (*32*). In the subsequent step, select subsets of metabolites and metabolite families, deemed to be of relevance based on the discovery experiments, will be precisely quantified in a targeted fashion by HILIC LC/MS using high-accuracy/high-resolution Orbitrap mass spectrometers (Q Exactive).

#### Transcriptional profiling (bulk RNA sequencing)

Transcriptional profiling is a powerful approach to identify biomarkers and mechanisms of immune-mediated diseases (*33*, *34*). Transcriptional profiling accurately reflects both dynamic changes in cellular composition and cellular response during the course of disease. Furthermore, network analysis approaches including cell deconvolution (*35*) and modular analysis (*36*) have been developed as robust computational approaches to unravel biologically coherent and insightful signatures across infectious and immunologic diseases and are particularly powerful approaches for longitudinal analyses (*37*–*40*). Transcriptional profiling of patients with COVID-19 has shown alterations in interferon responses and inflammatory pathways, which may relate to disease outcomes (*35*–*38*), sparking interest in immunomodulatory treatments directed at such pathways. The IMPACC network includes bulk transcriptomic analysis of upper (nasal, BRI Core Lab) and lower airway (EA—UCSF Core Lab) and PBMCs (Emory University and UCSF co-Core Labs).

#### Airway bulk RNA sequencing

RNA extracted from nasal and EA specimens is deoxyribonuclease (DNase)–treated, and human cytosolic and mitochondrial ribosomal RNA is depleted. Complementary DNA (cDNA) synthesis uses a random hexamer approach to capture human coding and noncoding RNA transcripts, as well as nonhuman RNAs. cDNA libraries are sequenced with paired-end reads at a target depth of 50 million reads per sample using NovaSeq S4 200 cycle flow cells. Human reads are aligned to the GRCh38 reference genome and quality-controlled by total counts per library and median coefficient of variation (CV) coverage. Raw counts for these genes are normalized across libraries according to the “Trimmed Means of M Values” (TMM) method (*41*), as implemented in the edgeR package for downstream analysis. Remaining nonhuman reads are aligned using the National Center for Biotechnology Information (NCBI) nucleotide and nonredundant protein databases followed by assembly of the reads matching each taxon detected. Repeated control and participant samples are sequenced within each sequencing batch to mitigate batch effects throughout the study.

#### PBMC bulk RNA sequencing

RNA is extracted from PBMCs lysed in Qiagen RLT buffer using the Zymo Quick-RNA MagBead Kit on an automated liquid handling system in batches designed to balance covariates across library preparation runs. Library preparation is performed using the TECAN NuGen Universal Plus mRNA-seq Kit in combination with the Qiagen FastSelect hemoglobin and ribosomal depletion kit to produce stranded, poly(A)–enriched mRNA-seq libraries depleted of ribosomal and hemoglobin RNA. Libraries are normalized using the output of shallow quality control (QC) sequencing runs and pooled using appropriate dilution ratios. The targeted read depth for each sample is at least 25 million reads per sample using NovaSeq S4 200 cycle flow cells and a 100–base pair, paired-end read length. Reads are aligned to a composite reference of the GRCh38 human genome and SARS-CoV-2 genome. Gene counts are generated internally with Spliced Transcripts Alignment to a Reference (STAR), and alignments are run through RNA-Seq by Expectation Maximization (RSEM) for comparison and alternate abundance metrics; in parallel, Kallisto will be run to produce transcript per million (TPM) values (*42*–*44*). Repeated measures of healthy control and participant samples are included to track intersite batch effects; Universal Human References RNA (UHRR) controls to assess intrasite variation, and reference PBMCs stimulated with Toll-like receptor 7 (TLR7) agonist will be included to assess intrasite batch sensitivity.

#### Mass cytometry—Blood

Single-cell technologies have provided techniques that can resolve disease in humans at an unprecedented level of detail, capturing the clinical and biological heterogeneity of disease. Mass cytometry or CyTOF uses rare metal isotope–conjugated antibodies for high-dimensional single-cell analysis. By using heavy metal ions as labels and detection in a TOF mass spectrometer, up to 50 single-cell parameters can be measured simultaneously with little/no background and minimal signal overlap between channels, providing unprecedented multidimensional cell profiling. CyTOF has been applied in suspension to characterizing immune cells in autoimmunity, cancer, and infection (*45*).

The CyTOF workflow implemented for this study has been designed with several specific considerations to reduce sample usage, streamline sample processing, and minimize experimental variability (*46*). Whole-blood samples are first stained at the site of collection using a commercial lyophilized 30-marker panel designed to identify all major circulating immune cell subsets (Fluidigm Maxpar Direct Immune Profiling Assay, see table S1) and are then fixed and cryopreserved (Smart Tube buffer). The cryopreserved samples are shipped to the IMPACC co-Core Labs at ISMMS and Stanford for barcoded batched processing, where they are labeled with a supplemental panel of 14 additional antibodies targeting fixation-resistant epitopes to resolve additional dynamic changes in cell phenotype. To maximize reproducibility, the supplemental panel has been formulated as a cocktail and frozen in single-use aliquots for each processing batch. The labeled barcoded samples are frozen for batched acquisition. The resulting FCS files are evaluated using a centralized data processing pipeline including bead-based sample QC and data normalization and automated sample demultiplexing.

#### Mass cytometry—EAs

CyTOF has recently been used to analyze cells in induced sputum, and numerous studies have validated this platform for multiparameter profiling of single cells from heterogeneous populations (*47*). The Yale Core Lab uses CyTOF on EAs of patients with COVID-19 to provide a higher-resolution understanding of the inflammatory responses in the affected tissue.

EAs from patients who require invasive mechanical ventilation are collected at the same time points as other samples. Saline is instilled (10 cm^3^) to collect the aspirate in a 40-cm^3^ Argyle specimen trap. To maximize cell viability, the aspirate is processed within 2 hours of collection. Cells are passed through a series of filters to isolate a single-cell suspension for labeling by CyTOF. For optimal detection of markers, surface antigens are labeled on fresh cells with a premade batch-prepared antibody cocktail before freezing at −80°C. To reduce variation, the remaining intracellular labeling is conducted on batches of samples together at the Yale University IMPACC EA CyTOF Core Lab. Antibody labeling of EA includes spiked-in reference cells (*48*) and markers to define polymorphonuclear leukocyte (PMN), monocytes, dendritic cells, natural killer cells, subsets of T and B lymphocytes, and 15 intracellular markers to quantify functional status (see table S2). CyTOF files from aspirates are normalized using the same centralized data processing pipeline and QC pipeline used for the CyTOF whole-blood samples, followed by a standard gating strategy for airway cells (*49*). EA samples are analyzed with CyTOF data from whole-blood samples of the same subjects, in concert with the other CyTOF Core teams.

#### Genomic analysis

The mechanisms by which an infection leads to severe disease in a subset of all infected individuals is incompletely explained. Immune responses to infection can differ based on both rare and common genetic variations (*50*). To identify any genomic determinants of severe COVID-19 disease, the Yale IMPACC Core Lab is conducting whole-exome sequencing (WES) and single-nucleotide polymorphism (SNP) genotyping and assessing genetic variants associated with individual susceptibility to severe disease. The DNA sequencing of IMPACC study subjects includes WES to include 19,433 genes that are in the RefSeq coding sequences, xGen exome capture, and whole-genome genotyping at 1.9 million SNP sites on the Illumina Infinium Global Diversity Array (GDA). For WES, genomic DNA will be extracted from frozen whole blood of each enrolled subject, with sample quality determined by spectroscopic and fluorometric methods. High-quality DNA will be sheared for automated library construction incorporating unique dual indices for each sample followed by hybridization-based enrichment of the exome. Pooled libraries will be sequenced on Illumina NovaSeq6000 S4 flow cells using optimized conditions for concentrations to maximize unique read output while limiting duplicates using paired-end sequencing chemistry and a read length of 101 bases. Following real-time analysis on Illumina’s CASAVA 1.8.2 software suite for converting signal intensities to individual base calls and completion of the run, raw data are evaluated for quality and samples are demultiplexed. Individual sample-level alignment to the human genome, variant calling, and annotation enable downstream analyses. Whole-genome genotyping will be performed following the manufacturer’s recommendations. Sequencing and array data will be available as FASTQ files to the analysis team accompanied by common variant association tests and rare variant gene burden tests for outcomes. Genetic sequence data will estimate population stratification and relatedness in our samples as covariates in other analyses.

## DATA MANAGEMENT AND ANALYSIS PLAN

### Study reporting and coordination

The study coordination and project management support of all participating IMPACC clinical, local laboratory processing, and Core research laboratory sites are centralized at the Clinical and Data Coordinating Center (CDCC) level (Fig. 4). A web-based clinical database system built on REDCap (*51*) (https://project-redcap.org) captures clinical data from all participants at all specified time points. CRFs harmonize with existing data standards put forward by the ISARIC, World Health Organization (WHO), and Prevention and Early Treatment of Acute Lung Injury (PETAL) Network. The clinical database contains participant- and visit-level identifiers to enable linkage with the sample tracking system and other immunophenotypic data. In coordination with the clinical information captured, a web-based sample tracking database LDMS (https://ldms.org) captures sample locations via barcode system in real time at point of collection. CDCC biostatistical support generates periodic reports of study enrollment and accrual, queries, summaries of primary study endpoints, final report, and any statistical tables, figures for scientific reports, and presentations. The CDCC provides training for each activity and serves as primary facilitator for intrastudy communications. This includes maintenance of a web-based communications portal to allow for individual- and group-based messaging and document sharing, a study pager to receive urgent text-based messaging, and coordination of all regularly scheduled working group meetings, which typically occur weekly, biweekly, or monthly. The CDCC provides continuous operational support, receiving and distributing any queries or concerns from study personnel related to logistics or study conduct aiming for near real-time resolution. Last, the CDCC developed and coordinates a network-wide patient newsletter to augment site strategies for participant retention.

### Collection of data and harmonization of data processing pipelines

Harmonized procedural and data processing pipelines across assays are coordinated with the Core Lab leads with the support of the Data Analysis Working Group (DAWG) and CDCC. As the cohort is recruited, plans will be made to address missing clinical data and samples and to appropriately randomize samples for each assay type. Once the data are uploaded to the IMPACC study data dashboard, CDCC data managers and biostatisticians will coordinate and verify QC processes for data collected/generated at clinical sites and Core Labs and will further perform additional quality assurance (QA) to maintain the highest possible accuracy of data before reporting and analysis.

### Centralized computing environment

The Amazon Web Services (AWS) (*52*) (https://aws.amazon.com) cloud computing platform is used for encrypted, access-controlled data storage and data analysis resources. A data and analysis dashboard allows IMPACC investigators to upload and store raw and processed computable data, and features a centralized computing environment enabling investigators to perform their analyses (*53*). Therefore, the AWS platform provides a computing environment for developing, testing, and running scripts, and performing QC and QA on the raw data generated by the Core Labs. This centralized computing environment also ensures that the IMPACC Core Labs use shared data standards, and are internally consistent, so that the datasets can be accurately analyzed in an integrated fashion. This approach is designed to facilitate data sharing and downstream analyses by IMPACC investigators and the broader research community, who will be able to access the data and associated metadata via the ImmPort public data repository (www.ImmPort.org).

### Clinical data analysis

Clinical outcomes will be defined for each patient in the study including binary outcomes (e.g., mortality yes/no) and disposition at day 14 or day 28 (e.g., discharged versus hospitalized versus deceased). Trajectory of clinical outcome will be defined at each time point that samples are collected using a seven-point ordinal scale modified from the WHO (https://who.int/blueprint/priority-diseases/key-action/COVID-19_Treatment_Trial_Design_Master_Protocol_synopsis_Final_18022020.pdf) and the NIAID eight-point ordinal scales (*54*) indicating disease severity, with clinical status defined as follows: (i) not hospitalized, no limitations; (ii) not hospitalized, activity limitations or requires home oxygen; (iii) hospitalized, not requiring supplemental oxygen; (iv) hospitalized, requires supplemental oxygen; (v) hospitalized, requires high-flow nasal cannula or noninvasive ventilation; (vi) hospitalized, requires invasive mechanical ventilation and/or extracorporeal membrane oxygenation (ECMO); and (vii) death. Latent class analysis or other unsupervised clustering techniques will determine categories of disease severity based on clinical outcomes or trajectories. Modeling of clinical data will associate symptoms, clinical characteristics, laboratory values, radiographic findings, and other experimental assay parameters at time of hospital admission and over the course of the disease that predict these categories of disease severity.

### Planned experimental data analyses

Core Lab assay data will be analyzed to identify correlates of clinical outcomes and COVID-19 disease severity and progression. Each Core Lab assay will be analyzed independently, and later, these data will be integrated to derive multiomic signatures and mechanistic hypotheses related to patient outcomes or COVID-19 disease trajectories. The initial analysis will be carried out using established pipelines for viral loads (*55*–*57*), viral sequencing (*58*, *59*), serology (*22*, *60*), serum cytokines (*61*, *62*), proteomics (*27*, *63*), metabolomics (*64*, *65*), transcriptional profiling (*36*, *39*, *66*), metagenomics (*67*, *68*), mass cytometry (*69*, *70*), and genomic (*71*, *72*) data. In general, low-dimensional assays, such as antibody titers and viral loads, will be analyzed directly, whereas high-dimensional data, such as RNA sequencing and mass cytometry, will first be projected into a lower-dimensional space for analysis. For genomic analysis, common variant association tests and rare variant gene burden tests will be conducted for outcomes, and genetic data will be used to estimate population stratification and relatedness in the IMPACC samples as covariates in other analyses.

The IMPACC study design offers a natural means to align the longitudinal samples collected from each patient with their hospital admission date. However, this approach may not be the most informative for identifying clinical associations. The comprehensive collection of clinical data will enable assessment of alternative approaches to align the samples, including time after onset of symptoms and/or time since escalation of care. The longitudinal nature of the IMPACC study will also allow exploration of disease trajectories for individual patients. Each of the derived measurements will be examined to determine whether they are coincident, lagging, or preceding indicators of disease severity.

The availability of a wide array of Core Lab assays probing different aspects of human immunity to SARS-CoV-2 on the same samples provides the opportunity for large-scale multiomic analysis. Jointly modeling the relations among different assay measurements allows for better understanding of the interactions driving the host response to SARS-CoV-2 and how it relates to clinical outcome. A supervised approach is used first to construct sparse factor models to predict clinical outcomes (per patient) or disease severity (per sample) from multiomic measurements at each time point (*73*–*76*), as well as for the longitudinal curves (*77*–*79*). Along with supervised analyses, unsupervised multiomic approaches will be used to define groups of patients that share similar immune profiles (*80*–*82*). This clustering can be done separately for each time point or by incorporating the full longitudinal time series for each patient (*36*, *83*–*85*). Once defined, the relationship between these clusters and clinical outcomes will be determined. This analysis will allow identification of both assay-specific and shared functional modules that explain the variability of clinical outcomes across patients. Identification of immunologic signatures can be validated with clinical data on disease severity and further refinement of clinical phenotyping, resulting in an iterative process to reveal immunologic mechanisms of disease and resulting clinical manifestations. As detailed below, data will be shared to leverage innovative analysis approaches from the wider scientific community.

### Governance

The IMPACC study leadership is composed of key program officers at NIAID, key members of the CDCC and DAWG, and study site principal investigators and clinical leads. This leadership group developed several key committees and working groups to oversee the project progress, resolve conflicts, ensure effective communication within the study team to achieve the study goals, and effectively disseminate IMPACC study results to the public. The governance infrastructure includes the following committees and working groups: The Steering Committee is responsible for the overall study oversight and conflict resolution; the Core Lab Working Group reviews core assay progress, QC, data analysis, and submission of the data to the CDCC; and the Clinical Working Group in collaboration with the CDCC reviews study progress including enrollment and retention, sample collection, and clinical data. Analysis and integration of the data is overseen by the DAWG in close collaboration with the Clinical and Core Lab Working Groups. The Publications Committee ensures compliance with the publications policy and reviews and prioritizes IMPACC manuscript proposals. In addition, the CDCC meets with clinical study coordinators and processing laboratory leads at recruiting sites weekly or biweekly to identify and mitigate barriers to successful study conduct. This committee/work group structure is essential to identify any obstacles or opportunities early on and to identify and deploy resources to meet the study objectives.

### Data sharing

IMPACC data sharing follows the NIH public data sharing policy (http://grants.nih.gov/grants/policy/data_sharing) to enable the widest dissemination of experimental and clinical data and associated metadata while also protecting the privacy of the participants and the utility of these datasets by deidentifying and masking potentially sensitive data elements. IMPACC will use the ImmPort database (*86*, *87*) (www.immport.org) as the primary database for experimental and clinical data and metadata dissemination. In addition, the IMPACC CDCC and DAWG leads will work with the ImmPort team to develop an IMPACC-specific resource page to facilitate public access to IMPACC datasets. Viral sequencing data will be deposited in GenBank (*12*, *88*) to provide access to the scientific community. The CDCC is responsible for deposition of clinical and Core Lab data and associated metadata to ImmPort, whereas deposition of all published site-specific datasets will be the responsibility of individual IMPACC sites. ImmPort will work closely with CDCC and specific sites to validate, curate, and verify submitted data and metadata. The IMPACC Publication Committee provides oversight for publications by establishing a uniform policy and transparent process for the preparation, prereview, and submission of IMPACC manuscripts and datasets.

## CONCLUSION

The goals of IMPACC are to longitudinally survey a large population of hospitalized patients with COVID-19 to inform disease progression dynamics and related biomarkers and conduct detailed, longitudinal immunophenotyping from presentation through disease progression or resolution. We aim to understand the interplay between viral load, immune pathology, and clinical manifestations of disease in a hospitalized COVID-19 cohort. Our study should improve understanding of the role of the host immune response in COVID-19 severity outcomes and help to generate hypotheses regarding effective host-directed therapeutics and optimal timing for administration of host response–directed interventions. The distinct features of IMPACC include leveraging of existing consortia and expert laboratories, efficient use of sample-sparing methods to maximize use of samples for multiple assays, and carefully harmonized data and sample collection, sample processing, and data sharing and analysis. Through this publication, we hope to facilitate coordination with other COVID-19 studies around the globe to foster data comparison and meta-analyses and to serve as a model for multicenter studies where a comprehensive, longitudinal assessment of biological processes is desired in the setting of public health emergencies.

## Supplementary Material

20210810-1

## Supplementary Materials

### This PDF file includes:

Methods: Sample Processing Manual of Procedures Tables S1 and S2

## Notes

View/request a protocol for this paper from *Bio-protocol*.
