# Supplementary material for: Immunophenotyping assessment in a COVID-19 cohort (IMPACC): A prospective longitudinal study
Source: Sci Immunol. 2021 Aug 10;6(62):eabf3733. doi: 10.1126/sciimmunol.abf3733 (PMC8713959; doi:10.1126/sciimmunol.abf3733)
Supplement: Supplementary file 2 — Methods: Sample Processing Manual of Procedures Tables S1 and S2 [file sciimmunol.abf3733_sm.pdf]

Science Immunology
AAAS

Supplementary Materials for

Immunophenotyping assessment in a COVID-19 cohort (IMPACC): A prospective longitudinal study

IMPACC Manuscript Writing Team on behalf of the IMPACC Network Steering Committee

Corresponding authors: Alison D. Augustine, augustine@niaid.nih.gov; Patrice M. Becker, patrice.becker@nih.gov

Sci. Immunol. **6**, eabf3733 (2021)
DOI: 10.1126/sciimmunol.af3733

The PDF file includes:

Supplementary Methods: Sample Processing Manual of Procedures
Table S1. Whole Blood CyTOF Panels
Table S2. Endotracheal Aspirate CyTOF Panels

Supplementary Methods: Sample Processing Manual of Procedures

1. Biological Sample Processing and Shipping

1.1 Biological Sample Processing Schematic

Study schedule

Biologic sample collection should be consistent with the clinical data collection and is conducted at study visits defined by the visit schedule schematic (Section 5.4.1). Baseline, including biospecimen collection is considered Visit 1 (within 48 hr of hospital admission). The remaining inpatient sample collection visits are Visit 2 (Day 4 ± 1 day), Visit 3 (Day 7 ± 1 day), Visit 4 (Day 14 ± 2 days), Visit 5 (Day 21 ± 2 days), Visit 6 (Day 28 ± 2 days if inpatient, -2/+7 days if outpatient). If the patient requires mechanical ventilation, the endotracheal aspirate (EA) will be collected on the same schedule.

Additional biologic samples (blood, nasal swab, endotracheal aspirates) will be collected if a participant requires escalation of care or is readmitted to the hospital more than 49 hours after discharge. These unscheduled visits should be scheduled within 24 hours and 96 hours of care escalation unless a study visit is already scheduled for one or both of these 24 and 96 hour windows.

Convalescent follow-up outpatient visits for Visit 7 (3 months), Visit 8 (6 months), Visit 9 (9 months), and Visit 10 (12 months) post hospital discharge (± 2 weeks). If a participant is discharged from the hospital prior to Visit 6 (Day 28), additional sets of biologic samples will be collected up to Visit 6, and the Day 28 visit window is extended.

Overall visual summary:

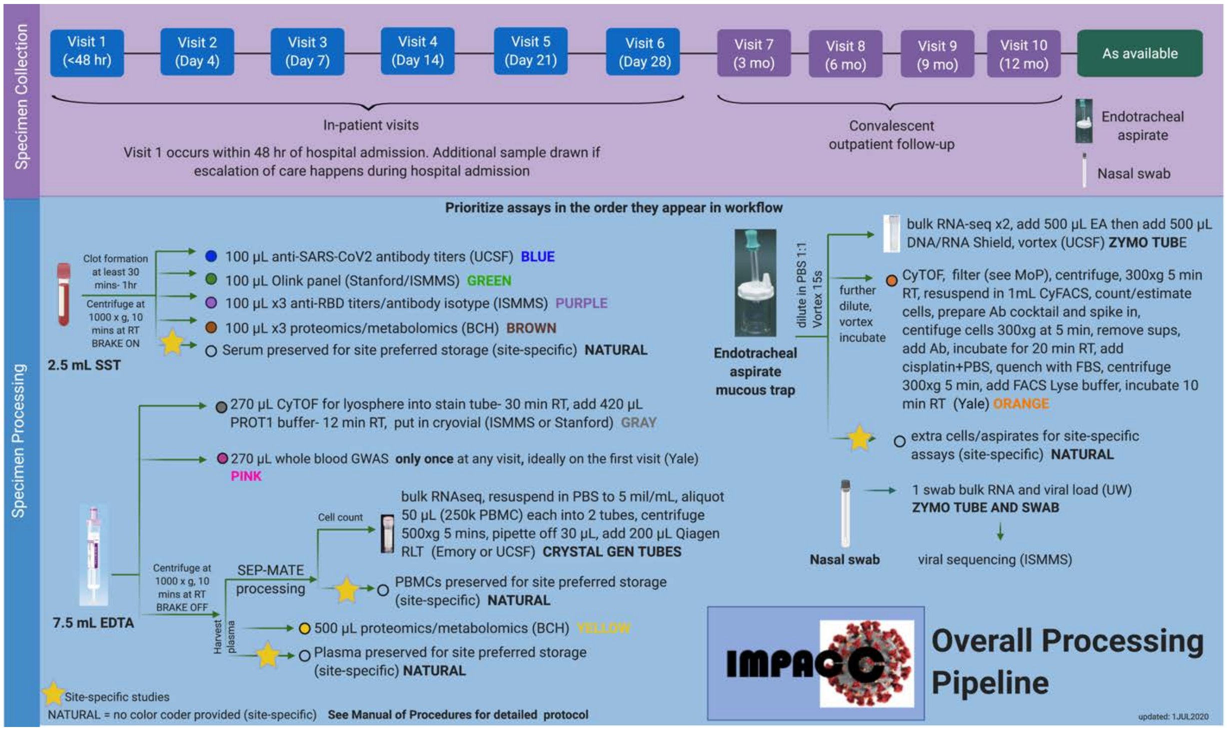

1.2 Serum Blood Processing: SST Tube

1.2.1 Sampling Schedule

Blood will be collected in one SST (2.5 mL Greiner SST tube) at all time points. **SST blood tubes need to be processed within 6 hours of collection.**

1.2.1 Visual summary

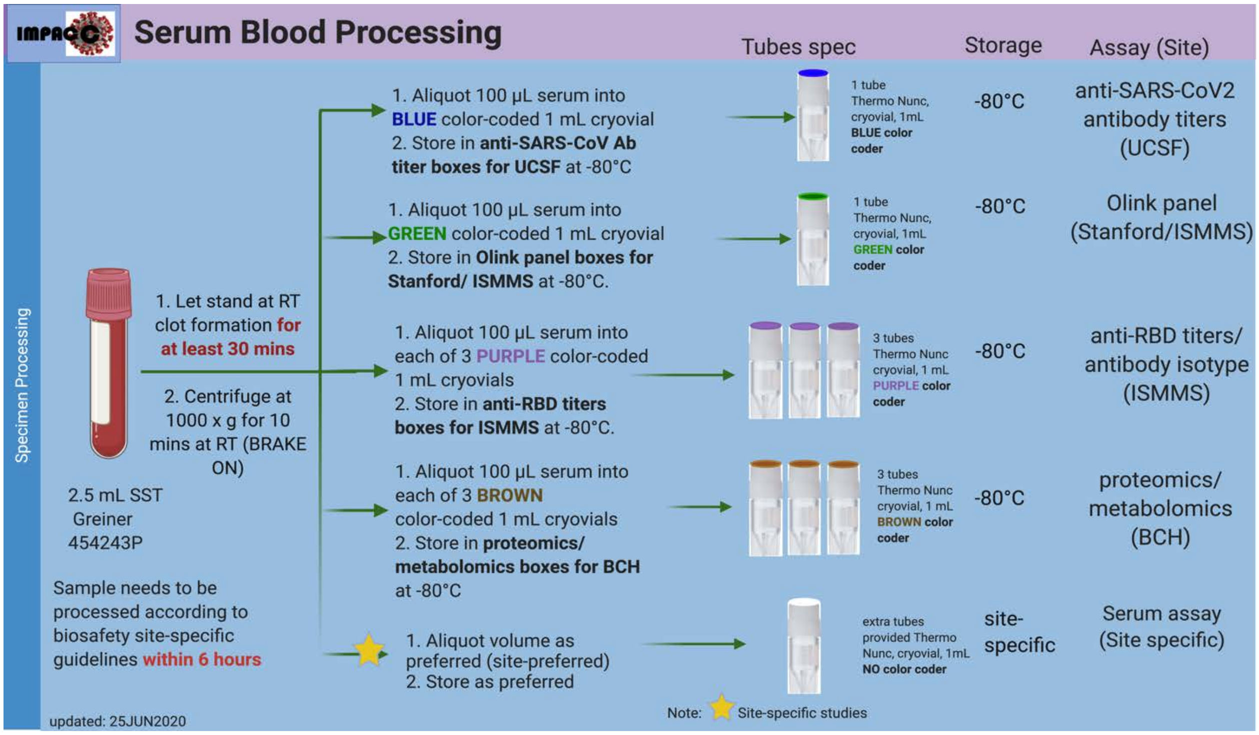

1.2.2 Materials

Clinical collection

- 2.5 mL Greiner Vacuette CAT Serum Separating Tube (Cat# 454243P)
- Serum Aliquots
    - 1.0 mL Thermo Nunc cryovials (Cat# 377224)
    - Color coder BLUE (Cat# 354879), GREEN (Cat# 355018), PURPLE (Cat# 375922), BROWN (Cat# 375868)
    - Tube labels
    - Storage boxes labeled to intended site
- Consumables (provided by each IMPACC study site)
    - Pipette tips

1.2.3 Protocol

1. Allow the SST tube to clot for at least 30 minutes at room temperature in a vertical position.
2. Prepare and label cryovials.
3. Centrifuge the serum-separating tube for 10 minutes at 1000 x g at room temperature (Swinging -out rotor and brake ON).

4. After centrifugation, the gel should be intact and the cells and serum completely separated. Do not re-centrifuge the tube if the barrier is incomplete.
5. Aliquot 100 µL serum into 1 mL cryovials and earmark **one** cryovial each with:
    a. **BLUE** color coder for **anti-SARS-CoV2 antibody titers for UCSF Serology Core Lab 1**
    b. **GREEN** color coder for **Olink Panel for your designated Olink Core lab (Stanford or Icahn School of Medicine at Mount Sinai (ISMMS))**.
6. Aliquot 100 µL into each of 1 mL cryovial (3x) and earmark three cryovials with*:
    a. **PURPLE** color coder for **anti-RBD titers/antibody isotype for ISMMS Serology Core Lab 2.**
    b. **BROWN** color coder for **serum proteomics/metabolomics for BCH Core lab.**
7. Place the samples at appropriate designated boxes and store at -80°C for shipment.
8. Remaining serum can be stored as preferred. We have provided two extra tubes. Boxes, extra cryovials and labels for the remaining serum samples are to be provided by the site.

If serum collected has a limited volume:

1. Aliquot 50 µL into one cryovial for **anti-SARS-CoV2 antibody titers for UCSF Serology Core Lab 1**
2. Aliquot 50 µL into one cryovial for **Olink Panel for your designated Olink Core lab (Stanford or ISMMS)**.
3. Aliquot 100 µL each into 3 cryovials for **anti-RBD titers/antibody isotype for ISMMS Serology Core Lab 2.**
4. Aliquot the remaining samples for **site-specific assays.**
5. No aliquot reserved **for serum proteomics/metabolomics for BCH Core lab**

1.3 Whole Blood, PBMC, Plasma Processing: EDTA Tube

1.3.1 Sampling Schedule

Blood will be collected in one monovette Sarstedt EDTA (7.5 mL) tube at all time points.
EDTA blood tubes need to be processed within 6 hours of collection.

Visual summary

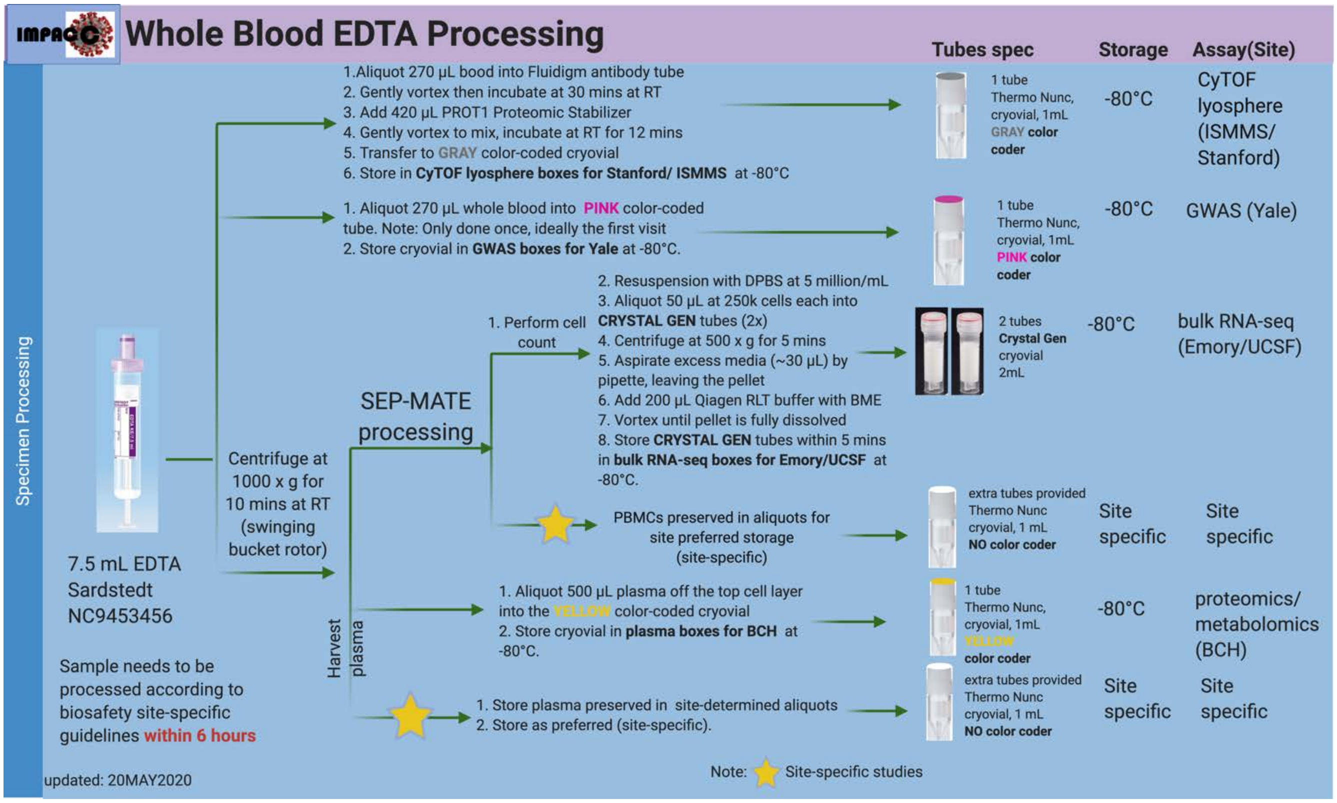

1.3.2 Materials

Clinical collection

- 7.5 ml Sarstedt Venous blood collection monovette EDTA (Cat# NC9453456)

1.3.2.1 Whole blood aliquots

- Crystal Gen tubes (Cat# 19335-6SPR)
- 1.0 mL Thermo Nunc cryovials (Cat# 377224)
- Color coders Pink, Yellow (Cat# 355077) and Gray (Cat# 375906)
- Antibody tube (part of Fluidigm Maxpar, provided by ISMMS/Stanford)
- Smart Tube Stabilizer from Smart Tube Inc (Fisher Sci. Cat# 501351692)
- Tube labels
- Storage boxes labeled to intended site

1.3.2.2 PBMC isolation

- SepMate-50IVD PBMC Isolation Tube (StemCell Cat #85460)
- SepMate-15IVD PBMC Isolation Tube (StemCell Cat #85420) (alternative if sample is <4 mL)
- Lymphoprep (StemCell Cat# 07861)
- EasySep Buffer (StemCell Cat# 20144)
- DPBS (Ca/Mg free; StemCell Cat #37354)

1.3.2.3 Reagents

- Qiagen RLT buffer (Cat #79216)
- Beta-mercaptoethanol

1.3.2.4 Consumables (provided by each IMPACC study site)

- 15 mL conical tubes or 50 mL conical tubes
- Disposable Transfer pipettes
- Pipette tips

1.3.3 Protocol:

1.3.3.1 If whole blood EDTA collected has total volume of less than 4.0 mL:

1. Proceed as normal for **CyTOF for ISMMS or Stanford.**
2. PBMC isolation will proceed using SepMate-15 isolation.
3. Plasma of 400 µL will be aliquoted for **metabolomics and proteomics for BCH.**
4. Blood for GWAS can be taken at another timepoint.

1.3.3.2 CyTOF sample preparation: for ALL time points

1. Invert EDTA tube to mix
2. Open a Maxpar Direct Immune Profiling Assay stain tube and label with sample information according to study protocol.
3. Working in a biosafety cabinet with appropriate PPE, remove the top of the blood tube.
4. Using a P1000 or equivalent pipetting device, add 270 µL blood to the labeled Maxpar Direct Immune Profiling Assay stain tube.
5. Cap the stain tube and gently vortex to mix.
6. Incubate at room temperature for 30 min.
7. In the biosafety cabinet, add 420 µL of PROT1 Proteomic Stabilizer.
8. Cap the stain tube and gently vortex to mix.
9. Incubate at room temperature for 12 min.
10. Transfer to labeled cryovial, earmark with color-coder as **GRAY**.
11. Place at assigned boxes for Whole blood CYTOF for your designated CyTOF Core lab (**Icahn School of Medicine at Mount Sinai (ISMMS)** or **Stanford)**. Store at -80°C for shipment.

Note: Stable storage at -80°C is very critical as temperature fluctuations of Smart-tube stabilized whole blood samples may cause clotting issues that will prevent analysis.

1.3.3.3 GWAS sample preparation: for only ONE time point

Note: Visit 1 (preferably, but can be another early time point)

1. Label and prepare cryovial.
2. Invert EDTA tube to mix
3. Working in a biosafety cabinet with appropriate PPE, remove the top of the blood tube.
4. Using a P1000 or equivalent pipetting device, aliquot 270 µL whole blood into a 1.0 mL cryovial.
5. Earmark with **PINK** color coder and store in **GWAS boxes for Yale Core lab**.
6. Immediately transfer to -80°C freezer.

1.3.3.4 Plasma sample preparation: for ALL time points

Centrifuge remaining blood sample at 1000 x g for 10 min at RT with swinging-out rotor and brake OFF.

1. Aliquot 500 µL plasma off the top cell layer in one **YELLOW** color coded cryovial.
2. Place at assigned boxes for **Plasma metabolomics/proteomics** for **Boston Children's Hospital** Core Lab. Store at -80°C for shipment.

1.3.3.5 Bulk RNA-seq preparation: for ALL time points

Note: If total volume of blood is more than 4.0 mL, use the PBMC isolation procedure using SepMate-50. If total volume of blood is less than 4.0 mL, use the PBMC isolation procedure using SepMate-15.

1.3.3.6 PBMC isolation (SepMate-50) (if total volume of blood is more than 4.0 mL)

1. Prepare SepMate-50 tube by pipetting 15 mL Lymphoprep below the insert, into the center hole
    a. Take care to minimize any air bubbles below the plastic divider
    b. Video from SepMate manufacturers (~2 min) https://www.youtube.com/watch?v=K9IlBxjgLt4
2. Dilute whole blood 1:2 with DPBS (Ca^{+2}/Mg^{+2} free)
    a. E.g. add 12 mL DPBS to 6 mL residual blood for a total volume of 18 mL
3. Slowly pipette the diluted blood down the side of the tube
    a. Some mixing may occur between the medium and blood, but take care not to mix under the divider
    b. Unlike conventional gradient separation, do not tilt the tube when adding diluted blood
4. Spin at 800 x g for 20 min at 20°C with brakes OFF
    a. Prepare 1 destination 50 mL tube (the yield will be ~16 mL per SepMate tube)
5. When the spin finishes gently pour the top layer into destination tubes.
    a. Do not invert the tubes for more than 2 seconds
    b. RBC contamination appears to be common in COVID patients. However, pipetting off the top layer from the SepMate tubes to avoid RBCs may cause significant lymphocyte loss, so pouring is recommended. RBC lysis is NOT recommended as it can damage the PBMCs.
6. Spin 400 x g for 10 min at RT with brake ON
    a. First spin at higher RCF because gradient medium is mixed with the cells.
7. Pour off supernatant, add EasySep buffer to 20 mL
8. Spin 300 x g for 10 min at RT with brakes ON
    a. Ensure that the supernatant is clear; opaque supernatant indicates incomplete centrifugation of cells. If necessary, perform the spin again at 400 g
9. Pour off supernatant, wash in 20 mL EasySep buffer for a second wash
10. Spin 300 x g for 10 min at RT with brakes ON

11. Pour off supernatant and resuspend in 5 mL DPBS
12. Proceed to Aliquoting for Bulk RNA-seq and Storage

1.3.3.7 PBMC isolation (SepMate-15) (if total volume of blood is less than 4.0 mL)

1. Prepare SepMate-15 tube by pipetting 4.5 mL Lymphoprep below the insert, into the center hole
    a. Take care to minimize any air bubbles below the plastic divider
    b. Video from SepMate manufacturers (~2 min) https://www.youtube.com/watch?v=K9IlBxjgLt4
2. Dilute whole blood 1:2 with DPBS (Ca/Mg free)
    a. For example, add 6 mL DPBS to 3 mL residual blood for a total volume of 9 mL
3. Slowly pipette the diluted blood down the side of the tube
    a. Some mixing may occur between the medium and blood, but take care not to mix under the divider
    b. Unlike conventional gradient separation, do not tilt the tube when adding diluted blood
4. Spin at 800 x g for 20 min at 20°C with brakes OFF
    a. Prepare 1 destination 15 mL tube (~8 mL per SepMate tube will be yielded)
5. When the spin finishes gently pour the top layer into destination tubes.
    a. Do not invert the tubes for more than 2 seconds
    b. RBC contamination appears to be common in COVID patients. However, pipetting off the top layer from the SepMate tubes to avoid RBCs may cause significant lymphocyte loss, so pouring is recommended. RBC lysis is NOT recommended as it can damage the PBMCs.
6. Spin 400 x g for 10 min at RT with brake ON
    a. First spin at higher RCF because gradient medium is mixed with the cells.
7. Pour off supernatant, add EasySep buffer to 20 mL
8. Spin 300 x g for 10 min at RT with brakes ON
    a. Ensure that the supernatant is clear; opaque supernatant indicates incomplete centrifugation of cells. If necessary, perform the spin at 400 x g
9. Proceed to Aliquoting for Bulk RNA-seq and Storage

1.3.3.8 Aliquoting PBMCs for Bulk RNAseq and Storage

Buffer Preparation: Prepare Qiagen RLT buffer and beta-mercaptoethanol (BME) at first use. Add 10 µl BME per 1 mL Buffer RLT. Dispense in a fume hood and wear appropriate protective clothing. Buffer RLT containing BME can be stored at room temperature for up to 1 month.

1. Pour off supernatant and resuspend in 5 mL DPBS.
2. Perform cell count.
    a. If RBC contamination will interfere with accurate PBMC counting, sites can perform RBC lysis on the counting aliquot ONLY (as per their lab's preferred

method). Please DO NOT perform RBC lysis on the entire sample, as it can damage the PMBCs.

3. Spin at 300 x g for 10 min
4. Resuspend in DPBS at 5 million per mL (add 1 mL for every 5 million cells)
5. Aliquot 50 µL (250,000 cells) each into CRYSTAL GEN tubes (2x) (if less than 5 million total cells, only aliquot 1 tube)
6. Centrifuge at 500 x g for 5 mins in a microfuge.
7. Carefully remove excess media (~30 µL) by pipette, leaving the pellet.
8. Add 200 µL QIAGEN RLT buffer with BME into each tube. Close cap. DO NOT MIX RLT & PELLET BY PIPETTING.
9. Vortex briefly until pellet is fully dissolved. If necessary to collect volume in bottom of tube, flick tube manually or perform 1 min spin in microfuge.
10. Place **CRYSTAL GEN** tubes (2x if enough cells) at assigned boxes for **bulk-RNAseq for your designated bulk RNAseq Core lab (Emory or UCSF**).
11. Store at -80°C for shipment ***within 5 min of preparation***. RNA is highly susceptible to degradation when left at room temperature.
12. The remaining PBMC samples can be aliquoted and preserved depending on site-preference. Two extra Thermo Nunc tubes are provided. Boxes, additional cryovials, freezing media and labels for the remaining cell samples are to be provided by the site.

1.4 Nasal Swabs

1.4.1 Sampling schedule

Nasal samples are collected once at all visits.

1.4.2 Visual Summary

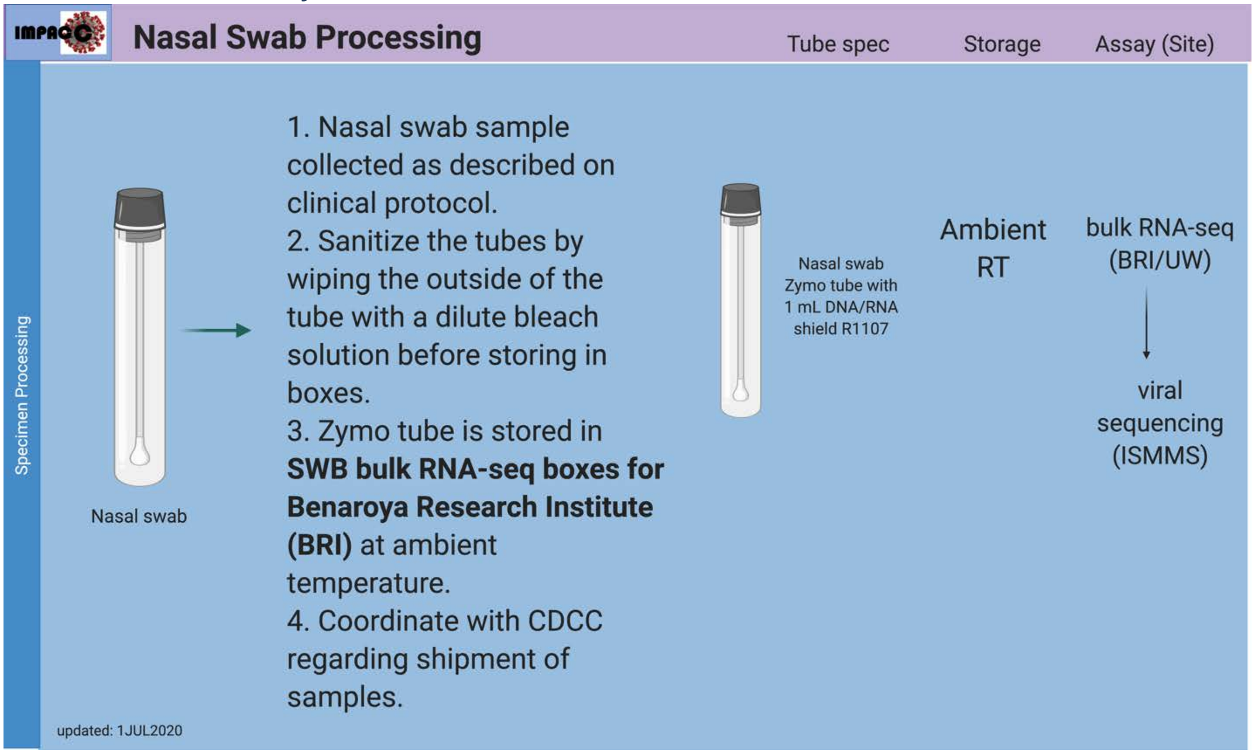

1.4.2 Materials

Collection Tube: 1ml DNA/RNA Preservative in a Polypropylene Screw-Cap Vial (Zymo Research, Cat#R1107)

Swab: with Polystyrene handle (Puritan Med)

Note

Sample is collected at clinical site. Benaroya Research Institute (BRI) will process the nasal swab samples. The samples will then be sent to University of Washington (UW) for bulk RNA sequencing. UW will be designated to ship all the samples to ISMMS for viral sequencing assays.

1.4.3 Protocol

1. Nasal swab sample on the tube brought from the clinical site should be assumed to have been collected as indicated in the clinical protocol.
2. Affix the label on the tube.
3. Sanitize the tubes by wiping the outsides of the tubes with a dilute bleach solution.
4. Please log the freezing time and date in LDMS.
4. Place the tube at designated boxes for Benaroya Research Institute (BRI) for Core Lab swab bulk RNA-sequencing and viral load assays.
5. Store at -80°C for shipment.

1.5 Endotracheal aspirate (EA) Processing

1.5.1 Sampling Schedule

Endotracheal aspirate will be collected in one 40 cc Argyle specimen traps at all time points (for intubated patients only). **Endotracheal aspirate samples need to be processed within 2 hours of collection.**

1.5.2 Visual summary

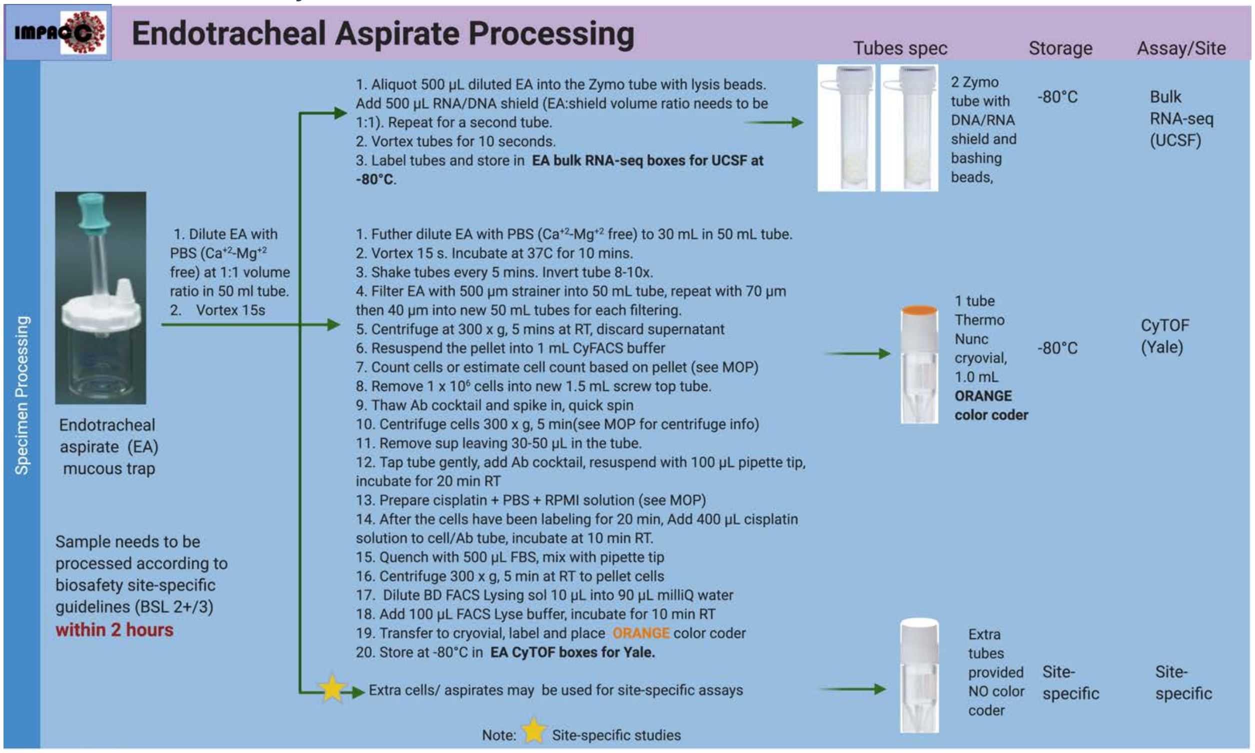

1.5.3 Materials

Clinical collection

- 40 cc Argyle specimen traps (Cat# 8884724500)

EA sample processing/filtering

- Cell strainer 70 µm white (Cat # 087712)
- Cell strainer 40 µm blue (Cat # 087711)
- pluriSelect pluriStrainer 500 µm (Cat # NC0822591)
- 1.5 mL screw top O-ring, sterile tube (Cat # 230820)

EA storage tubes and materials

- Zymo Research Bashing Beads Lysis Tubes (S6012-50)
- 1.0 mL Thermo Nunc cryovials (Cat# 1265168N)
- Color coder **ORANGE** (Cat# 355158)
- Tube labels
- Storage boxes labeled to intended site

Reagents

- Zymo DNA/RNA Shield Buffer (provided in 1L buffer/ site)
- Maxpar PBS (Cat #201058)
- Maxpar Cell Staining Buffer (CyFACS) (Cat #201068)
- Cell-ID Cisplatin (Cat# 201064)
- BD FACS Lysing Solution 10 (Cat 349202)
- RPMI + Glutamine (Cat #1460)
- Maxpar Water (Cat# 201069)
- Antibody cocktail tube (provided by Yale group)
- Fetal bovine serum (provided by Yale group)

Consumables (provided by each IMPACC study site)

- Pipette tips
- 50 mL Falcon conical tubes
- 10 mL pipettes

1.5.4 Protocol

Endotracheal aspirates processing workflow summary

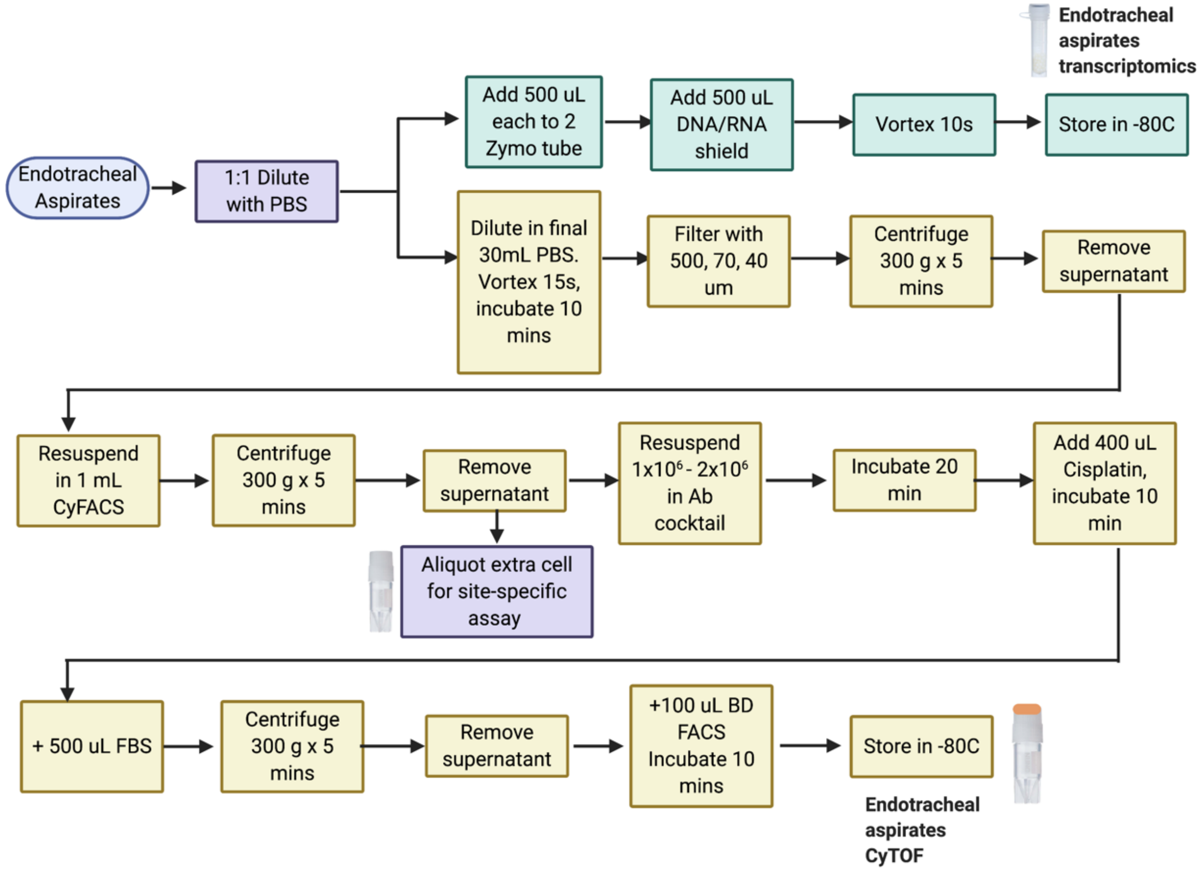

Note: Refer to your Biosafety lab guidelines while processing endotracheal aspirates. **Endotracheal aspirate samples need to be processed within 2 hours of collection.**

Buffer Preparation: On the first thaw of the cisplatin stock, aliquot it out with 20 µl per tube and store at -20C for later use.

1.5.4.1 If endotracheal aspirates collected has limited volume:

1. Aliquot only 1 tube for **UCSF for bulk RNAseq.**
2. Proceed with CyTOF processing for the **Yale** Core Lab.
3. Aliquot the remaining samples (if any) for site-specific assays. Note: Intubated patients usually have their tube flushed several times a day as part of clinical care. If additional sample is needed for site-specific studies, site staff can coordinate with their clinical team to obtain another EA sample.

1.5.4.2 EA sample processing and RNA sample collection

1. Dilute EA with Maxpar PBS (Ca^{+2}-Mg^{+2} free) at 1:1 ratio in 50 ml tube.
2. Vortex 15s.
3. Aliquot 500 µL diluted EA into the Zymo tube with lysis beads and add 500 µL of DNA/RNA Shield (the key part is that the EA:Shield ratio needs to be 1:1). Repeat for a second tube.
4. Vortex tubes for 10 seconds.
5. Label tubes and store in **EA bulk RNA-seq for UCSF** at -80°C.

1.5.4.3 For CyTOF EA samples

1. Further dilute EA with Maxpar PBS (Ca^{+2}-Mg^{+}2 free) to 30 ml in 50 ml tube.
2. Vortex 15s then incubate at 37°C for 10 min. Shake tube every 5 min.
3. Invert tube gently 8-10 times.
4. Filter EA liquid sequentially through 3 filters using a 10 ml pipette (gravity flow filtration):
   a. Using a 10 ml pipette transfer EA liquid through 500 µm filter into a new 50ml tube.
   b. Using a 10 ml pipette transfer EA liquid through a 70 µm filter into a new 50ml tube.
   c. Using a 10 ml pipette transfer EA liquid through a 40 µm filter into a new 50 ml tube.
5. Centrifuge at 300 x g for 5 min at RT to pellet the cells.
6. Remove and discard the supernatant.
7. Resuspend the cell pellet into 1 ml Maxpar Cell Staining buffer (CyFACS).
8. Count the cells [or optional estimate cell number from size of pellet – please see below

1.5.4.3.1 Cell counting procedure if using automated cell counter:

1. Take out 10 µl of EA cell suspension and mix well with 10 µl of Trypan blue.
2. Add the mixture into the lacune of a cell counting slide and insert slide into counter. (NOTE: This is a general protocol for automated cell counter, you should follow your own facility instruction in cell/trypan blue ratio and calculation formula)
3. Record the reading, calculate the volume of suspension which contain 1 x 10^6 cells.

4. Remove the volume of 1 x 10^6 cell suspension into 1.5 ml screw top tube. (Optional: Extra cells can be stored by the site for future use).
5. Thaw antibody cocktail tube, quickly spin down tube to collect liquid at the bottom of the tube.
6. Centrifuge cells at 300 x g for 5 min at RT to pellet the cells. [If not using a horizontal rotor centrifuge, rotate the tube 180 degrees and spin briefly to bring the pellet to the bottom.] Remove and discard the supernatant leaving about 30-50 µl in the tube.
7. Tap tube gently to resuspend the cell pellet, add antibody cocktail and mix gently with 100 µl pipette tip. Try to avoid making bubbles. Skip to STEP 10 below (7.5.4.4).

1.5.4.3.2 *Alternative to Cell Counting: estimate the cell number from pellet size:* Put 5ul, 10 µl and 15 µl of trypan blue into 3 screw cap tubes, put it in hood as your reference to estimate cell pellet size of 1, 2 and 3 million cells, respectively. Follow the instructions below.

1. Thaw antibody cocktail tube, quickly spin down tube to collect liquid at the bottom of the tube.
2. Centrifuge cells at 300 x g for 5 min at RT to pellet the cells. [If not using a horizontal rotor centrifuge, rotate the tube 180 degrees and spin briefly to bring the pellet to the bottom.] Remove and discard the supernatant leaving about 30-50 µl in the tube.
3. Estimate cell number from size of pellet compared to the size of the trypan blue tubes.
4. Discard the supernatant and leave about 30-50 µl in the tube to resuspend the cells. Before resuspending the cells in the supernatant, examine the pellet size to estimate cell number:
    - o If the cell pellet size is around 1 million or less (size of 5 µl trypan blue or smaller), directly go to STEP 9 below (7.5.4.4).
    - o If cell pellet size is around 2 million (size of 10 µl trypan blue), resuspend the cells in the remaining buffer by tapping the tube gently and split half of the cell suspension out of the tube and save it for site specific study.
    - o If cell pellet size is around 3 million (size of 15 µl trypan blue), resuspend the cells in the remaining buffer by tapping the tube gently and split 2/3 of the cell suspension out of the tube and save it for site specific study.
    - o NOTE: staining 1 x 10^6 is optimal, but fewer cells can also provide good results. Staining more than 2 x 10^6 cells will significantly decrease the quality of the CyTOF results.
5. Add antibody cocktail to the “resuspended” cells and mix gently with 100 µl pipette tip. Try to avoid making bubbles. Skip to STEP 10 below (7.5.4.4) (for samples that were estimated to have greater than 1 X 10^6 cells)

1.5.4.4 Proceed here after cell counting:

9. Tap tube gently to resuspend the cell pellet, add antibody cocktail and resuspend with 100 µl pipette tip. Try to avoid making bubbles.
10. Incubate at RT for 20 min.
11. Prepare 100 µM Cisplatin by diluting 1 µl of cisplatin stock (5 mM stock) in 50 µl Maxpar PBS. Add 20 µl of 100 µM cisplatin to 380 µl RPMI+L for final concentration of 5 µM.
12. After the cells have been labeling for 20 min, add 400 µl cisplatin (5 µM solution) directly into the cells/antibody tube. Mix with pipette tip. Incubate at RT for another 10 min (not longer!).
13. Quench by adding 500 µl FBS. Mix with pipette tip.
14. Centrifuge at 300 x g for 5 min at RT to pellet the cells.
15. Dilute BD FACS Lysing Solution 10 µl into 90 µl milliQ water.
16. Remove and discard the supernatant.
17. Add 100 µl BD FACS Lysing Solution, resuspend with pipette tip. Incubate at RT for 10 min to fix.
18. Transfer into 1 mL Thermo Nunc vial. Label and earmark the cryovial with **ORANGE.**
19. Store at -80°C in **EA CYTOF boxes for Yale**.
20. The remaining EA samples can be aliquoted and preserved depending on site-preference. Boxes, extra cryovials, freezing media and labels for the remaining EA samples are to be provided by the IMPACC site.

Table S1. Whole Blood CyTOF Panels

Core Fluidigm MDIPA panel (labeled at site of blood collection)			
Isotope	Target	Clone	Source
89Y	CD45	HI30	Fluidigm
141Pr	CCR6	G034E3	Fluidigm
143Nd	CD123	6H6	Fluidigm
144Nd	CD19	HIB19	Fluidigm
145Nd	CD4	RPA-T4	Fluidigm
146Nd	CD8a	RPA-T8	Fluidigm
147Sm	CD11c	Bu15	Fluidigm
148Nd	CD16	3G8	Fluidigm
149Sm	CD45RO	UCHL1	Fluidigm
150Nd	CD45RA	HI100	Fluidigm
151Eu	CD161	HP-3G10	Fluidigm
152Sm	CCR4	L291H4	Fluidigm
153Eu	CD25	BC96	Fluidigm
154Sm	CD27	O323	Fluidigm
155Gd	CD57	HCD57	Fluidigm
156Gd	CXCR3	G025H7	Fluidigm
158Gd	CXCR5	J252D4	Fluidigm
160Gd	CD28	CD28.2	Fluidigm
161Dy	CD38	HB-7	Fluidigm
163Dy	CD56	NCAM16.2	Fluidigm
164Dy	TCRgd	B1	Fluidigm
166Er	CD294	BM16	Fluidigm
167Er	CCR7	G043H7	Fluidigm
168Er	CD14	63D3	Fluidigm
170Er	CD3	UCHT1	Fluidigm

Supplemental antibodies (labeled at IMPACC Core labs)			
Isotope	Target	Clone	Source
111Cd	GranzymeB	REA226	Miltenyi
112Cd	IgA	Polyclonal	Southern Biotech
113In	CD11b	M1/70	Biolegend
114Cd	IgM	MHM-88	Biolegend
115In	CD69	FN50	Biolegend
116Cd	IgG	Polyclonal	Southern Biotech
142Nd	ICOS	C398.4A	Biolegend
159Tb	CD39	A1	Biolegend
162Dy	CD169	7-239	Biolegend
165Ho	CD64	10.1	Biolegend
169Tm	CD71	CY1G4	Biolegend
175Lu	PD-1	EH12.2H7	Fluidigm
209Bi	CD61	VIPL2	Fluidigm

171Yb	CD20	2H7	Fluidigm
172Yb	CD66b	G10F5	Fluidigm
173Yb	HLADR	LN3	Fluidigm
174Yb	IgD	IA6-2	Fluidigm
176Yb	CD127	A019D5	Fluidigm

Table S2. Endotracheal Aspirate CyTOF Panels

Isotope	Target	Clone	Source
89Y	CD45	HI30	Fluidigm
141Pr	CD3	UCHT1	Fluidigm
142Nd	TMPRSS2	polyclonal	ThermoFisher
143Nd	HLA-DR	L243	Fluidigm
144Nd	CD69	FN50	Fluidigm
145Nd	CD4	RPA-T4	Fluidigm
146Nd	CD8a	RPA-T8	Fluidigm
147Sm	CD20	2H7	Fluidigm
148Nd	Eotaxin	43915	R&D
149Sm	CD127	A019D5	Fluidigm
150Nd	MIP-1b	D21-1351	Fluidigm
151Eu	CD123	6H6	Fluidigm
152Sm	TNFa	Mab11	Fluidigm
153Eu	CD62L	DREG-56	Fluidigm
154Sm	CD45	HI30	Fluidigm
155Gd	ACE-2	535919	NOVUS
156Gd	IL-6	MQ2-13A5	Fluidigm
158Gd	IFNg	B27	Fluidigm
159Tb	CD11c	Bu15	Fluidigm
160Gd	CD14	M5E2	Fluidigm
161Dy	CD80/B7.1	2D10.4	Fluidigm
162Dy	CD66b	80H3	Fluidigm
163Dy	CD56	NCAM16.2	Fluidigm
164Dy	CD15	W6D3	Fluidigm
165Ho	CD61	VI-PL2	Fluidigm
166Er	Cytokeratin	C-11	BioLegend
167Er	CD11b	ICRF44	Fluidigm
168Er	CD206	15-2	Fluidigm
169Tm	CoV2 Spike protein	1A9	GeneTex
170Er	CD54	HA58	Fluidigm
171Yb	CD68	Y1/82A	Fluidigm
172Yb	EPX	MM82.2.1	Mayo Clinic
173Yb	IL-8	E8N1	BioLegend
174Yb	IL-1b	H1b-27	BioLegend
175Lu	IFN-b	IFNb/A1	BioLegend

176Yb	Siglec-8	837535	R&D
209Bi	CD16	3G8	Fluidigm

Green: labeled at site of sample collection (surface lineage markers)
Pink: labeled at Yale Core lab (intracellular markers)
